# Choropleth map legend design for visualizing the most influential areas in article citation disparities: A bibliometric study

**DOI:** 10.1097/MD.0000000000017527

**Published:** 2019-10-11

**Authors:** Tsair-Wei Chien, Hsien-Yi Wang, Chen-Fang Hsu, Shu-Chun Kuo

**Affiliations:** a Medical Research Department, Chi-Mei Medical Center; b Department of Sport Management, College of Leisure and Recreation Management, Chia Nan University of Pharmacy and Science; c Ncphrology Department, Chi-Mei Medical Center; d Department of Partiatrics, Chi-Mei Medical Center, Yong Kang; e Department of Optometry, Chung Hwa University of Medical Technology, Jen-Teh; f Department of Ophthalmology, Chi-Mei Medical Center, Yong Kang, Tainan City, Taiwan.

**Keywords:** choropleth map, Gini coefficient, Google Maps, legend design, Pubmed Central

## Abstract

**Background:**

Disparities in health outcomes across countries/areas are a central concern in public health and epidemiology. However, few authors have discussed legends that can be complemental to choropleth maps (CMs) and merely linked differences in outcomes to other factors like density in areas. Thus, whether health outcome rates on CMs showing the geographical distribution can be applied to publication citations in bibliometric analyses requires further study. The legends for visualizing the most influential areas in article citation disparities should have sophisticated designs. This paper illustrates the use of cumulative frequency (CF) map legends along with Lorenz curves and Gini coefficients (GC) to characterize the disparity of article citations in areas on CMs, based on the quantile classification method for classes.

**Methods:**

By searching the PubMed database (pubmed.com), we used the keyword “Medicine” [journal] and downloaded 7042 articles published from 1945 to 2016. A total number of 41,628 articles were cited in Pubmed Central (PMC). The publication outputs based on the author's x-index were applied to plot CM about research contributions. The approach uses two methods (i.e., quantiles and equal total values for each class) with CF legends, in order to highlight the difference in x-indices across geographical areas on CMs. GC was applied to observe the x-index disparities in areas. Microsoft Excel Visual Basic for Application (VBA) was used for creating the CMs.

**Results:**

Results showed that the most productive and cited countries in *Medicine (Baltimore)* were China and the US. The most-cited states and cities were Maryland (the US) and Beijing (China). Taiwan (x-index = 24.38) ranked behind Maryland (25.97), but ahead of Beijing (16.9). China earned lower disparity (0.42) than the US (0.49) and the rest of the world (0.53) when the GCs were applied.

**Conclusion:**

CF legends, particularly using the quantile classification for classes, can be useful to complement CMs. They also contain more information than those in standard CM legends that are commonly used with other classification methods. The steps of creating CM legends are described and introduced. Bibliometric analysts on CM can be replicated in the future.

## Abstract 2

Supplemental Digital Content is available in the text

**Key points**

- CM legend was particularly emphasized, highlighted, and designed in this study, which was rarely reported in previous publications.
- The area-based academic achievements can be measured by the authorship-weighted scheme, which was published in previous articles.
- The way to create CMs, particularly shown on Google Maps, has been demonstrated through an mp4 video. This is a worthy reference for bibliometric analyses on CM in the future.

## 1 Introduction

The earliest known choropleth map (CM) called cartes teintées (colored map in French) was created by Baron Pierre Charles Dupin in 1826.^[1]^ The term “choropleth map” was coined by John Kirtland Wright in 1938.^[2]^ The most famous illustration of CM was applied to the results of the 2000 US presidential election.^[3]^ Recently, many examples of disparities in health outcomes across areas, such as dengue outbreaks,^[4,5]^ disease hotspots,^[6]^ and the Global Health Observatory (GHO) maps on major health topics,^[7]^ have been presented.

Although CMs give a good visual impression of disparity across areas, there are certain disadvantages that can be experienced when using them. For example, they do not directly show the associated statistical distribution of the data.^[8]^ In addition, using CMs makes it very difficult to distinguish proportional frequencies between different shades.^[9]^ To resolve these problems, 2 approaches (i.e., CM with ogive-based legends or proportional symbols) have been proposed.^[8–10]^ However, the distribution of the data regarding value disparities across classified classes has not been effectively and entirely solved so far. Thus far, the Gini coefficients (GCs)^[11]^ and the Lorenz curve^[12]^ have yet to be used in explaining such phenomena. The Lorenz curve plots the cumulative frequency (CF) of averages across classes against the equality line, that is, the 45-degree from the left-bottom to the right-top point, which is equivalent to the CF of counts in classes if the quantile classification method is applied). After all, the Lorenz curve has become a standard method for analyzing health inequalities and disease patterns across geographic units.^[13–15]^ This is because the index of disparities (e.g., Gini) measures the evenness with a variable, such as the dengue infection rates against 10,000 population (also called “density”), which is distributed across a set of classes, such as dengue outbreaks. The GC index, which ranges from 0 (perfectly uniform) to 1.0 (perfectly concentrated within a class), indicates whether or not occurrences are concentrated in a particular class.^[16]^

In economics, the GC is a measure of statistical dispersion intended to represent the income or wealth distribution of a nation's residents; it is also the most commonly used measurement of inequality regarding numbers of observations across classes. Usually, residents’ incomes are ranked in ascending order and are divided into 5 groups with an equal number in size based on a nation's residents. The alarming level of GC for denoting inequality is set at 0.4.^[16–18]^

Bibliometric analyses have been frequently applied in recent years.^[19–23]^ We are thus interested in using the CM technique in analyzing a scholarly journal to investigate country-based citation disparities around the world. In this study,

(1) the Lorenz curve and GCs are applied to report the characteristics of value legends on CMs,
(2) two classification methods of the quantile (i.e., equal count each class) and the vital few (i.e., equal total values in classes) are compared, and
(3) the CM with symbols (i.e., using 1, 2, 3 to highlight the vital few) are demonstrated for visualizing the most influential areas using the disparities of article citations.

## 2 Methods

### 2.1 Data source

By searching the PubMed database (pubmed.org), we used the keyword “Medicine” [Journal] on April 7, 2019, and downloaded 7,042 articles published from 1945 to 2016. An author-made Microsoft Excel Visual Basic for Application (VBA) module was used to analyze and present the research results. All downloaded abstracts were based on the type of journal article. All data used in this study were downloaded from Pubmed Central (PMC). This means that ethical approval is not necessary for the study, in accordance with the regulation promulgated by the Taiwan Ministry of Health and Welfare.

### 2.2 Classification methods

Five types of classification methods have been proposed in the literature^[24,25]^ and 3 to 7 data classes have been suggested to simply highlight geographic areas.^[26]^ Some are those like the known-class method (1) (e.g., political maps in the US with only 2 classes, the well-known red state/blue state maps) and the equal interval method (2) (i.e., dividing the data into equal range classes), such as ages in 0 to 10, 10 to 20, 20 to 30, etc, which are spread across the entire range. We avoided using equal intervals if data were skewed to one end. Those outliers, in that case, would likely produce empty classes for wasting classes with no observations in them.

The quantiles (3) tend to create attractive maps that place an equal number of observations in each class.

The problem with quantiles is that value legends with classes have very different numerical ranges (e.g., 1–4, 5–9, 10–250…the last class is huge): a situation that is very undesirable. The advantage of using quantiles is that they are suitable for GC and analysis of variance (ANOVA) owing to the equal number of sizes among classes.

Natural breaks (4) (e.g., using an algorithm as k-mean^[27]^) is a kind of optimal classification scheme, which finds class breaks that—for a given number of classes—will minimize within-class variance and maximize between-class differences. Meanwhile, the manual method (5) manually sets a rule for setting class breaks. For example, we hope to select the vital few classes using the equal total values in each class, thus resulting in the highest vital class with the fewest observations.

We will demonstrate several abovementioned methods, using data of the US regions and the state population to display the CM.

### 2.3 Two types of legends designed for CM

The CFs using (1) total values, (2) count numbers, and (3) the averages across all classes were particularly designed in order to complement the traditional legend with only a single legend with class cutting points on CM. In addition, the links for yielding the GCs and Lorenz curve were present on another legend for providing more information on the disparities of (1) total values, (2) count numbers, and (3) the averages across classes.

### 2.4 Two types of Lorenz curves designed for CM

We designed 2 types of Lorenz curves that display the 2 classification methods (i.e., equal counts and equal total values in each class). This was done in order to complement the CM for visualizing the disparities of article citations among classes. We also included all authors in the term of x-index > 0 in order to calculate the GCs and define them using the formula^[18]^ 
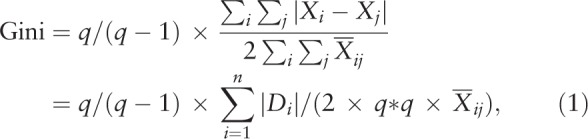


where *D* is the absolute difference of each pair data element between classes, Xij is the data element value, Xij-bar is the mean of all values across classes, *q* is the number of classes, and *q*/(*q*-1) is the adjustment for the data on the number of classes to reach 1.0 (i.e., an extreme inequality). Otherwise, the extreme GC reaches 0.8 using the Eq. 2 when *q* is 5,^[18]^ as shown below. 
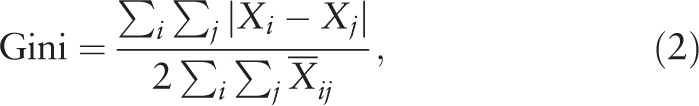


### 2.5 The most productive and cited countries/areas in publications

The x-index^[28]^ is determined by the formula 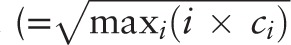, Eq. 3), where all the number of cited papers (denoted by ci) are based on the maximum multiplied by *i* and *ci* when citations are in descending order. The contribution weight (*Wj*) for each coauthor in an article byline is defined using the formula (Eq. 4)^[18]^ 
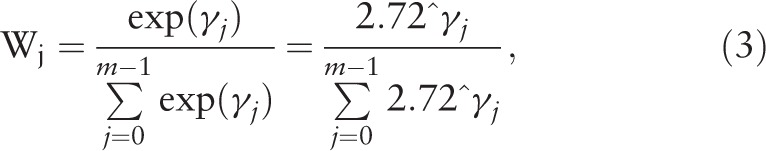


The variable *W*_*j*_ in Eq. 3 denotes the weight for an author on the order j in the article byline. The power γ_*j*_ is an integer number from *m*-1 to 0 in descending order. The author number is *m*-1. The first author gains the highest credit and follows by the last being the corresponding author.

The most productive and cited countries/areas in publications were shown on a world-based CM. The disparities were further analyzed using GCs and Lorenz curves and then displayed on a country-based CM. The country-based (or province-/state-based) x-indices were obtained by sorting all authors’ contribution weights first in descending order and then calculating the maximal root of *ci* multiplied by the article position at *i*, as shown in Eq. 3.

### 2.6 Video abstract for organizing data

We provide readers with Lorenz curves under four scenarios, in order to understand the features of GCs (see Supplemental Digital Content 1). One mp4 video (Supplemental Digital Content 2) presented the research process of extracting articles and citations from PMC to make the CM using MS Excel VBA. A dataset regarding this study was included in Supplemental Digital Content 3. Hopefully, anyone who is interested in our work can replicate our research procedure in the future.

## 3 Results

### 3.1 Types of classes on CM about the legends

We present 4 types of classification methods:

(1) known classes for 10 US regions,
(2) equal intervals for the US state population,
(3) quantiles for equal number counts across states in each class, and
(4) equal total values for each class for selecting the vital few (e.g., the top three) on the CM.

**Figure 1:**
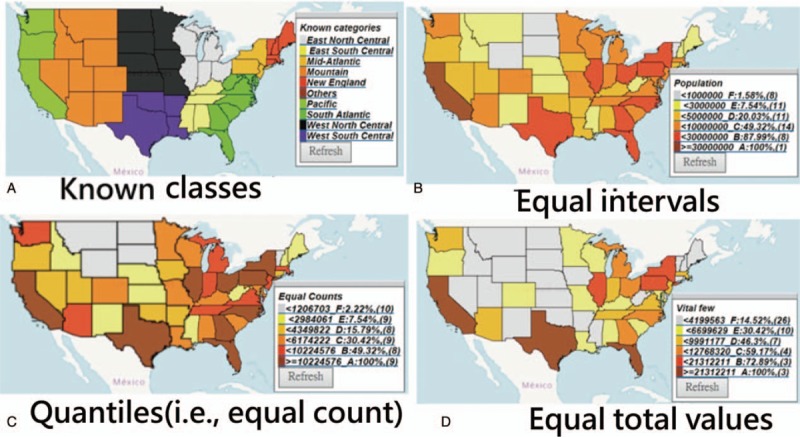
Types of choropleth maps on the classification methods and the legends.

The reader may refer to the legends on the accumulative frequency and the number of observations for each class shown in Figure 1.

### 3.2 The most productive and cited counties/areas on publications in *Medicine*

#### 3.2.1 The most productive countries in Figure 2

**Figure 2:**
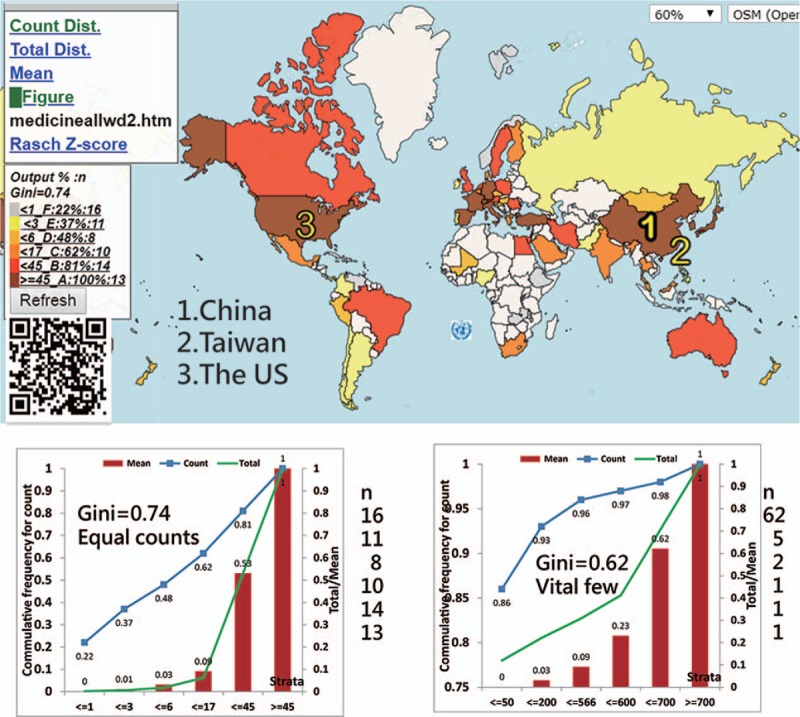
Article outputs in comparison with those found around the world.

The most productive counties/areas on publications in *Medicine (Baltimore)* were China, Taiwan, and the US (see Fig. 2).

The most productive authors were Chia-Hung Kao (Taiwan) with an author impact factor (AIF) of 3.18 = 75.76/23.79 = citations/the citable and x-index = 6.02), Sung Ho Jang (South Korea, AIF = 1.70 19.94/11.71 and x-index = 3.16), and Manuel Ramos-Casals (Spain, AIF = 24.72 = 190.45/7.70, and x-index = 9.4).

#### 3.2.2 The most cited countries in Figure 3

**Figure 3:**
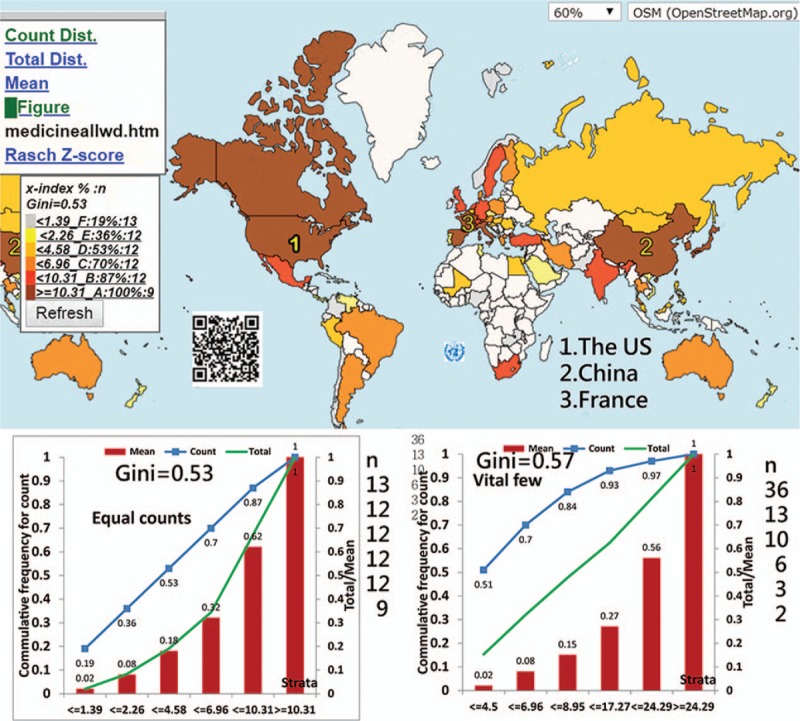
Using the x-index to compare achievements around the world.

The most-cited counties/areas were the US, China, and France (Fig. 3) represented by the symbols 1, 2, and 3, respectively.

The top 3 authors who have the highest x-index were JA Winkelstein (the US, 12.8), MJ Chusid (the US, 10.21), and CJ Epstein (the US, 10.12).

#### 3.2.3 The proper legends necessarily combined with choropleth maps

Both Lorenz curves (i.e., the left panels beneath the CM in Figs. 2 and 3) show that the disparities in publication outputs (0.74) are higher than the cited (0.53) ones based on the quantiles. The 2 right-side Lorenz curves in the classes are less meaningful because their counts in classes are unequal.

#### 3.2.4 Other complemental legends provided to readers

Aside from those legends (i.e., marked with GC, cutting points, accumulative frequencies, and counts for each class), we also provide other plots with Lorenz curves (e.g., counts, total values, and the mean for classes) through links that redirect to the websites when clicked. Interested readers are suggested to scan the QR codes on Figures to see CMs in detail on Google Maps.

### 3.3 The most influential states/provinces in article citations

**Figure 4:**
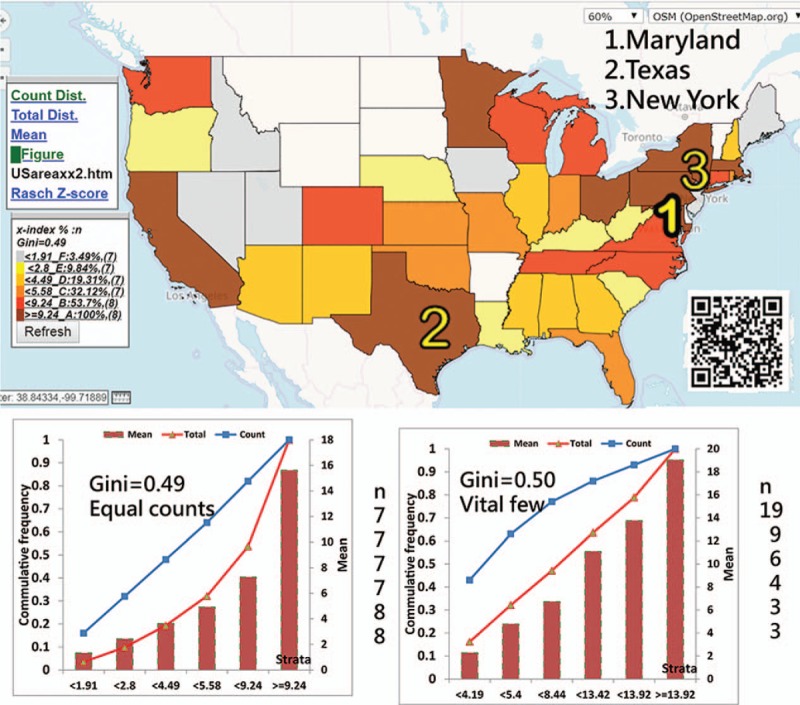
Using the x-index to compare achievements in the US.

**Figure 5:**
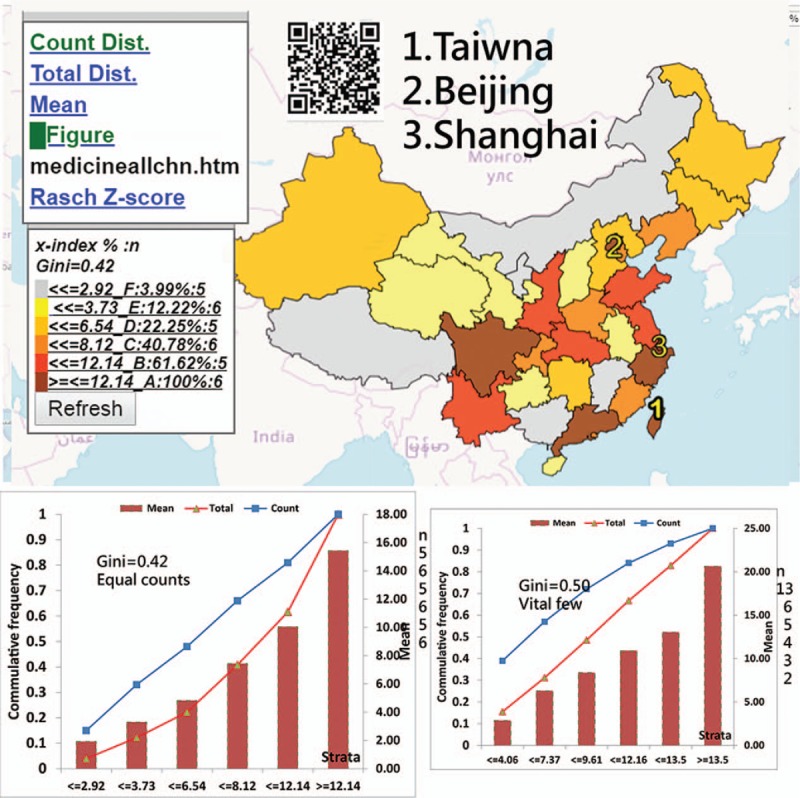
Using the x-index to compare achievements in China.

The most-cited states (or provinces) are Maryland in the US and Beijing in China. Taiwan (x-index = 24.38) is lower than Maryland (25.97) but higher than Beijing (16.9). China earned a lower disparity (0.42) than the US (0.49) and the rest of the world (0.53) when the GCs are applied (Figs. 4 and 5, respectively).

## 4 Discussions

We found that the most productive and cited countries in *Medicine (Baltimore)* are China and the US, respectively. The most-cited states (or provinces) are Maryland in the US and Beijing in China. China earned a lower disparity (0.42) than the US (0.49) and the rest of the world (0.53), all of which are beyond the alarming level of GC (0.4) for denoting inequality.^[16–18]^

Many articles displayed disparities in health outcomes across areas or regions, such as dengue outbreaks,^[4,5]^ disease hotspots,^[6]^ or even the GHO maps on major health topics,^[7]^ using traditional equal intervals, which is a method that is considered useful when distribution of the data has a rectangular shape in the histogram.^[29]^ However, datasets following uniform distributions that make all accumulative frequencies be equivalent using the four abovementioned classification methods (i.e., equal intervals, quantiles, equal total values, and equal average values in each class, see Supplemental Digital Content 4) are rare in the real world. The main disadvantage of this equal interval classification is that it fails to consider how data are distributed along the equality line,^[30]^ as shown in the Lorenz curve in Figure 2. If a different classification (e.g., quantiles) with a percentage legend (e.g., Figures in this study) is used, the data information about the disparity could be shown more effectively on the CM.

According to previous studies^[8,10]^ introducing ogive-based legends with accumulative frequencies for CM is not as clear nor as useful as our proposed method using Lorenz curves and the two legends in comparing disparities among classes (see Figures).

Classification methods for CMs have already been proposed in the literature.^[24–26]^ However, thus far, no such type of equal total values has been illustrated in academic articles. The advantage of using equal total values in each class is that it can clearly determine and highlight the vital few. As shown in Figures 2 to 4, the symbols from 1 to 3 on CM can highlight the top three on the map. According to the advice on the website,^[9]^ even the CM with proportional symbols or the graduated size of the circle within each area^[31]^ can be easily made if the layering technique on a map can be applied, which is what we did in the Figures or as those on the website.^[32]^

The most productive author is Chia-Hung Kao (Taiwan), who published 149 papers related to using national health database in 2015.^[33]^ His research achievement (i.e., AIF = 3.18 and x-index = 6.02 based on a specific journal or a topic) is disclosed in this study.

The first feature of this study is that it used the quantile classification method that allows the accumulative frequency to be equivalent to both the average value and the total values (TV) in each class. That is because the average = TV/*ni*, where all values of *ni* (or the total observation counts/the number of classes) are equal. As such, the Lorenz curve can be correctly plotted, as shown in the respective plot on the left bottom panel in Figures.

The second feature is to apply (1) the adjusted Gini formula (i.e., Eq. 1), (2) the x-index calculation (i.e., Eq. 3), (3) the author weight equation (i.e., Eq. 4), and (4) the CMs to evaluate data disparities and author individual research achievements (IRA), which are seldom discussed in previously published articles. The reason, without considering h-index as the main metric, is discussed in examples.^[28]^ Hence, the x-index has a higher discrimination power in IRA than the h-index.

The third feature is that this study used PMC citations. We found over 100 papers with a search of “most-cited articles” [Title] in PMC on April 4, 2019. For example, 5 articles^[19,23,34–36]^ were found in *Medicine (Baltimore)*. Most applied academic databases, such as the Scientific Citation Index (SCI; Thomson Reuters, New York, NY), Scopus (Elsevier, Amsterdam, the Netherlands), and Google Scholar,^[37,38]^ to investigate the most-cited articles in a specific discipline. In comparison, none used the PubMed library (i.e., a free search engine accessing primarily the MEDLINE database of references and abstracts on life sciences and biomedical topics) to retrieve the citing articles.

Despite the findings shown above, several potential limitations require further research efforts in the future. First, this study only addressed one target journal; it should be generalized to other fields in the future.

Second, there might be some biases when identifying author names because different authors with the same name or abbreviation but affiliated with different institutions (or states/provinces) exist.

Third, we recommend using quantile classification for determining classes on CM, which distributes a set of counts into classes containing an equal number of counts. It would be impossible for the counts in each class to be totally equal using the quantile method. The almost-equal number of observations might slightly affect the feature of the GC, which requires all classes to be nearly equal in size.

Fourth, although our suggestions are limited to both legends and the Lorenz curves using GCs to display on CM, other techniques and skills on CM, such as determining the number of data classes, the color progression, and software used for preparing the CMs, are not included in this study. Interested readers are recommended to read other relevant references in the literature. The CM made in this study has been recorded in an mp4 video (see Supplemental Digital Appendix 2). Details on all coordinates on Google Maps can be seen by scanning the QR code on the Figures and right-clicking the mouse on the web page to read the original HTML.

Finally, in order to ensure that the quantiles are effective and useful on legends, we included in the analysis only those areas with x-indexes that are greater than zero. Different samples yield disparate results, such as GCs. Future researchers should take note of this in their works.

## 5 Conclusion

CF legends and complemental plots, such as Lorenz curves, are recommended to be used in preparing CMs. The quantile classification is meaningful in processing the calculated GCs. In this work, we illustrated examples containing more information than those in standard CM legends that are commonly used with any other classification methods. The steps involved in creating CM legends are introduced to bibliometric analysts for applications in the future.

## Acknowledgments

We thank Enago (www.enago.tw) for the English language review of this manuscript.

## Author contributions

**Conceptualization:** Tsair-Wei Chien.

**Data curation:** Hsien-Yi Wang.

**Formal analysis:** Hsien-Yi Wang, Chen-Fang Tsai Hsu.

**Investigation:** Shu-Chun Kuo.

**Methodology:** Tsair-Wei Chien.

**Project administration:** Shu-Chun Kuo.

**Resources:** Chen-Fang Tsai Hsu, Shu-Chun Kuo.

**Software:** Tsair-Wei Chien.

**Supervision:** Shu-Chun Kuo.

**Validation:** Tsair-Wei Chien, Chen-Fang Tsai Hsu.

**Visualization:** Hsien-Yi Wang.

**Writing – original draft:** Hsien-Yi Wang.

Tsair-Wei Chien orcid: 0000-0003-1329-0679.

## Supplementary Material

Supplemental Digital Content

## Supplementary Material

Supplemental Digital Content
