# Supplementary material for: Choropleth map legend design for visualizing the most influential areas in article citation disparities: A bibliometric study
Source: Medicine (Baltimore). 2019 Oct 11;98(41):e17527. doi: 10.1097/MD.0000000000017527 (PMC6799475; doi:10.1097/MD.0000000000017527)

Additional file 2: The study process for making choropleth maps

http://www.healthup.org.tw/marketing/course/marketing/chorplethmaps.mp4


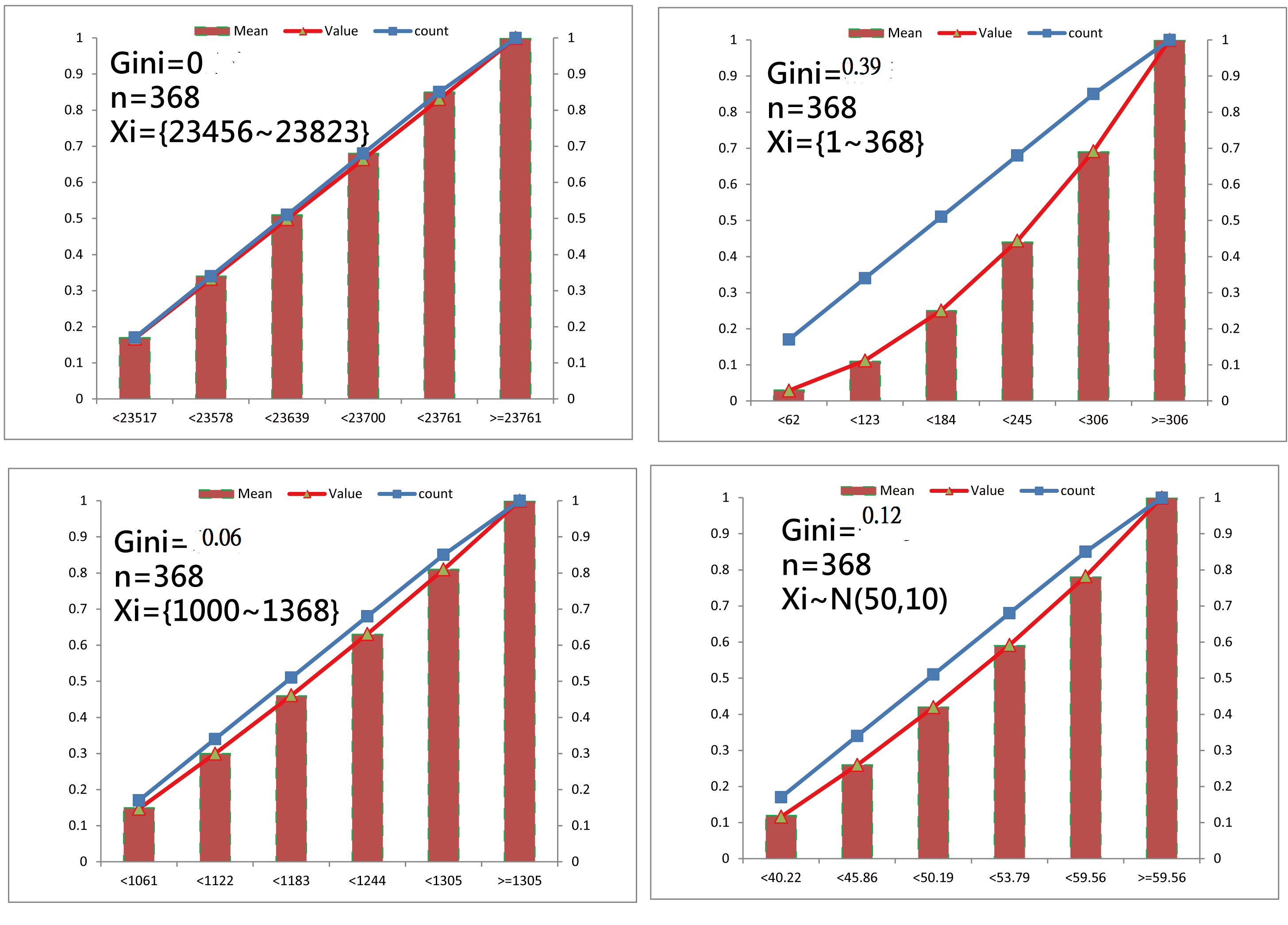


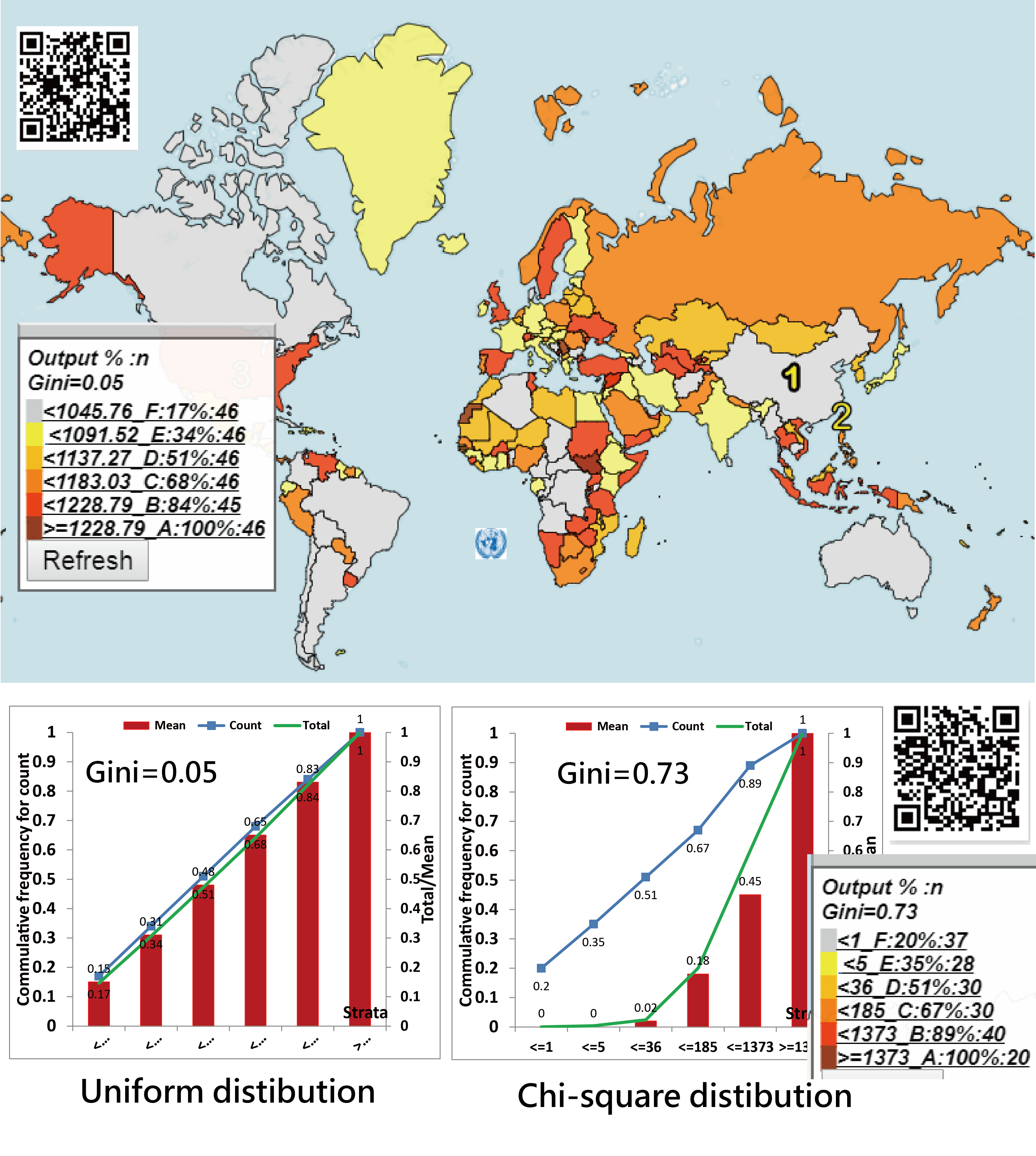

Supplement: Supplemental Digital Content [file medi-98-e17527-s002.doc]
